# Prevalence of nasal colonisation by methicillin-sensitive and methicillin-resistant *Staphylococcus aureus* among healthcare workers and students in Madagascar

**DOI:** 10.1186/s12879-016-1733-6

**Published:** 2016-08-15

**Authors:** Benedikt Hogan, Raphael Rakotozandrindrainy, Hassan Al-Emran, Denise Dekker, Andreas Hahn, Anna Jaeger, Sven Poppert, Hagen Frickmann, Ralf Matthias Hagen, Volker Micheel, Sabine Crusius, Jean Noel Heriniaina, Jean Philibert Rakotondrainiarivelo, Tsiriniaina Razafindrabe, Jürgen May, Norbert Georg Schwarz

**Affiliations:** 1 Research Group Infectious Disease Epidemiology, Bernhard Nocht Institute for Tropical Medicine (BNITM), Bernhard-Nocht-Str. 74, D-20359 Hamburg, Germany; 2 Department of Microbiology and Parasitology, University of Antananarivo, B.P. 175, Antananarivo, Madagascar; 3 University Medical Center, Hamburg-Eppendorf, Martinistr. 52, 20251 Hamburg, Germany; 4 Department of Tropical Medicine at the Bernhard Nocht Institute, German Armed Forces Hospital of Hamburg, Bernhard-Nocht-Str. 74, D-20359 Hamburg, Germany; 5 Institute for Medical Microbiology, Virology and Hygiene, University Hospital of Rostock, Schillingallee 70, D-18057 Rostock, Germany

## Abstract

**Background:**

Methicillin-resistant *Staphylococcus aureus* (MRSA) clones pose a significant threat to hospitalised patients because the bacteria can be transmitted by asymptomatic carriers within healthcare facilities. To date, nothing is known about the prevalence of *S. aureus* and MRSA among healthcare workers in Madagascar.

The objective of our study was to examine the prevalence and clonal epidemiology of nasal *S. aureus* and MRSA among healthcare workers and non-medical University students in Antananarivo, Madagascar.

**Methods:**

This cross sectional study screened nasal swabs taken from students and healthcare workers for *S. aureus*. Multiplex PCR was performed to identify *S. aureus*-specific (*nuc*), MRSA-specific *mecA* and *mecC* genes, Panton-Valentine leukocidin (PVL) (lukF-PV), and toxic shock syndrome toxin-1 (TSST-1) specific genes in methicillin-sensitive *S. aureus* (MSSA) and MRSA isolates. *Staphylococcus* protein A gene (*spa*) typing was performed for all confirmed MRSA isolates. The frequency distribution of nasal *S. aureus* and MRSA of healthcare workers and non-medical students was compared using Pearson’s χ^2^ test.

**Results:**

Of 1548 nasal swabs tested, 171 (11 %) were positive for *S. aureus*; 20 (1.3 %) of these isolates were identified as MRSA. *S. aureus* was detected in 91 of 863 healthcare workers (10.4 %) and in 80 (11.8 %) of 685 students; however, 14 (1.5 %) healthcare workers carried MRSA compared with six (0.9 %) students. Nasal carriage of *S. aureus* and MRSA was more prevalent in women than in men, and 21 (11.7 %) *S. aureus* isolates were PVL-positive and 36 (21 %) were TSST-1 positive. The *mecC* gene was not detected in any isolates. Five different *spa* types were identified, with *spa* type t186 being the predominant MRSA clone (16/20).

**Conclusion:**

The results of the present study reveal a low frequency of *S. aureus* and MRSA nasal carriage in both students and healthcare workers from Antananarivo, Madagascar. The predominant MRSA clone (t186) was previously described in hospitalised patients in Madagascar.

**Electronic supplementary material:**

The online version of this article (doi:10.1186/s12879-016-1733-6) contains supplementary material, which is available to authorized users.

## Background

The bacterium *Staphylococcus aureus* causes a variety of infections, ranging from skin and soft tissue infections to life-threatening disease [1]. It is also a commensal that colonises the anterior nares of asymptomatic carriers, who may unknowingly transmit the pathogen within the community or within healthcare facilities [2]. Carriage among healthcare workers (HCW) may act as a source of infection in hospitals [3]. Since the introduction of *β*-lactam antibiotics, the spread of methicillin-resistant *S. aureus* (MRSA) has increased on a global scale [4, 5].

Data regarding the prevalence and distribution of methicillin-sensitive *S. aureus* (MSSA) and MRSA in Africa are scarce, and control measures within healthcare settings are limited due to constraints with respect to resources and diagnostic facilities [6]. Studies on *S. aureus* carriage by hospital patients in Madagascar report a MRSA prevalence of between 4 and 13 % [7–11]. The prevalence of *S. aureus* nasal carriage in outpatients was 38, and 15 % of cases were MRSA [7]. Madagascan hospitals set no guidelines for screening patients or HCW for MRSA.

Different MRSA strains have emerged in hospitals and in the community in both Europe and the USA [12, 13]. Factors such as travel and close contact with animals may contribute to the dissemination of the bacterium in Africa: one MRSA clone in particular (ST-88-MRSA), which is found worldwide, is also widespread in West, Central, and East Africa [14]. A multicentre study conducted across five different African countries identified one major clone, which is also predominant in Madagascar (ST-88 IV, *spa* type t186) [15]. A high prevalence of the staphylococcal virulence factor, Panton-Valentine leukocidin (PVL), was found in MSSA strains isolated from hospital patients from mainland Africa and the Western Indian Ocean region [8, 16, 17]. Evidence suggests that PVL is associated with staphylococcal skin and soft tissue infections [18]. No data are available on the presence of PVL in MSSA and MRSA isolates obtained from HCW in Madagascar. Another staphylococcal virulence factor, toxic shock syndrome toxin-1 (TSST-1), causes severe illness and multisystem clinical manifestations [19]. Few studies report the prevalence of TSST-1 in healthy carriers from Africa; to date, no data are available with respect to Madagascan HCW [20, 21]. Resistance to *β*-lactam antibiotics (as in MRSA) is due to the acquisition of the *mecA* gene. In recent years, a similar gene termed *mecC* has been described, which is often not detected by routine laboratory tests because standard molecular methods usually focus only on detecting *mecA* [22].

Here, we obtained nasal swabs from HCW from different hospitals and from healthy non-medical students at the University of Antananarivo. The aim was to identify the prevalence and local clonal epidemiology of *S. aureus* and MRSA in Madagascar.

## Methods

### Study design

This cross sectional study enrolled non-medical students from the University of Antananarivo and HCW from five different hospitals (University Children’s Hospital, the surgery department at the University Gynaecological Hospital, the University Infectious Diseases department, a military hospital, and a private clinic for general and trauma surgery) and one dispensary in Antananarivo, Madagascar. HCW were defined according to the WHO definition [23], which included all study participants working in a hospital. Hospital staff were informed and recruited during in-house training sessions, while university students were asked to participate during seminars and lectures.

### Sample size calculation

The required sample size to yield at least k = 5 MRSA isolates with about 90 % probability was estimated by assuming that the proportion of MRSA among *S. aureus* isolates was 5 %, and that 35 % of all sampled individuals were carrying *S. aureus*. Using the formula

$$ 1-{\displaystyle \sum_{i=0}^k\left({}_k^n\right)}{p}^k{\left(1-p\right)}^{n-k} $$

the required minimum sample size was estimated at 526 per arm (HCW and non-medical university students).

### Data and sample collection procedures

After providing informed consent, demographic data (e.g., sex, age, home community) was collected via a questionnaire. Students were asked to name their faculty, and hospital employees were asked to describe their role and whether their work included regular contact with patients. Respondents were also asked about hospital admissions within the last 6 months, their history of antimicrobial treatment within the last 6 months, whether they lived in a student/healthcare staff hall, whether they nursed chronically ill relatives at home, whether they suffer from a chronic illness, and whether they had any acute skin diseases such as atopic dermatitis, psoriasis or chronic ulcers. As *S. aureus* colonises pets and livestock, the participants were asked whether they had contact with animals [14]. After the participants filled in the questionnaire, they were instructed on how to collect a self-administered nasal swab.

### Microbiological examination of nasal swabs in Madagascar

The study team demonstrated the technique used to obtain nasal samples using a sterile cotton-tipped swab (Copan Nasal Swab Transsystem®, Brescia, Italy) and provided assistance where necessary. The samples were then immediately transported to the study laboratory where bacteria were isolated as described below.

After incubation on Columbia blood agar (OXOID®, Vienna, Austria) for 18–24 h at 35–37 °C, the first step of the identification procedure involved examination of colony morphology and Gram staining. Suspected *S. aureus* colonies on the blood agar plate were tested for catalase and coagulase activity, and for latex agglutination (Staphytec plus, OXOID®) [24]. *S. aureus* strains were tested for antibiotic susceptibility using the standard disc diffusion method on Mueller-Hinton Agar, according to current CLSI (Clinical and Laboratory Standards Institute) guidelines. Antibiotic susceptibility testing was done for the following locally available and prescribed antibiotics: penicillin, erythromycin, clindamycin, cotrimoxazole, and ampicillin/sulbactam. Cefoxitin was used as an MRSA screening agent.

*S. aureus* ATCC-25923, BAA 9176, and methicillin-resistant *S. aureus* ATCC-43300 were used as controls. *S. aureus* isolates were stored at −80 °C in Cryobank tubes (Cryobank, MAST®, Liverpool, UK) until shipment at -20 °C to the Bernhard Nocht Institute for Tropical Medicine, Germany, for molecular analyses. The QIAamp DNA Mini Kit (Qiagen®, Hilden, Germany) was used to extract DNA from all *S. aureus* isolates, according to the manufacturer’s instructions.

### Multiplex PCR to confirm *S. aureus* isolates

In Germany, all *S. aureus* isolates were further screened for *S. aureus*-specific (*nuc*), MRSA-specific (both *mecA* and *mecC*), and PVL and TSST-1 virulence genes by multiplex PCR. The primers used are listed in Additional file 1: Supporting Information Table S1.

PCR was performed using the Qiagen® multiplex PCR kit (ref. no.-201643). Only strains harbouring the *mecA* or *mecC* genes were classified as MRSA [25].

### *Staphylococcus* protein A gene (*spa*) typing of MRSA isolates

To examine the clonal epidemiology of MRSA, all PCR-confirmed MRSA isolates were subjected to *spa* typing using the RidomStaphType standard protocol, followed by double-strand sequencing [26]. Sequences were analysed using RidomStaphType version 2.2.1 software (Ridom Ltd., Würzburg, Germany). Associated multi-locus sequence types (MLST) were automatically allocated by the Ridom SpaServer according to the based-upon-repeat-pattern (BURP) algorithm (if sufficient data were available) [27], or retrieved from the literature.

### Treatment of MRSA-positive individuals

If the nasal swabs tested positive for MRSA in Madagascar, the participant was offered treatment with Mupirocin (Turixin®) ointment to eradicate the nasal MRSA (treatment followed the guidelines set down by the Robert-Koch-Institute, Germany) [28]. After treatment, further nasal swabs were taken to confirm successful decolonisation.

### Statistical analyses

Pearson’s χ^2^ test or Fisher’s exact test was used to compare the frequency distribution of *S. aureus* and MRSA nasal carriage by HCW and non-medical students. Age, sex, occupation, and previous antimicrobial use were variables entered into a multivariable logistic regression model to assess their adjusted association with *S. aureus* carriage. Analyses were performed using Stata 12 (StataCorp LP, College Station, USA) software.

## Results

**Fig. 1:**
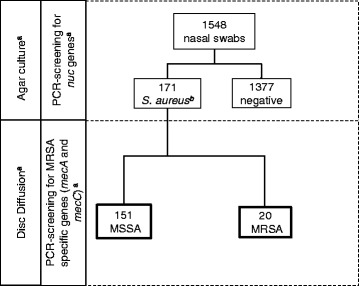
Examination procedure for nasal swabs obtained from university students and healthcare workers. In a first step *S.aureus* were identified via PCR-screening for *nuc* genes and in a second step these *S.aureus* were screened for *mecA* and *mecC* genes allowing to differentiate between methicillin-resistant and methicillin-sensitive *Staphylococcus aureus*

Nasal swabs were taken from 863 HCW and 685 students. *S. aureus* was identified in 171 isolates (11.0 %), of which 20 (1.3 %) were MRSA (Fig. 1).

The point prevalence of nasal *S. aureus* carriage was 91 (10.4 %) in the HCW group and 80 (11.4 %) in the student group. MRSA carriage was slightly higher in the HCW group (1.5 %) than in the student group (0.9 %).

Of the MSSA isolates, 7.3 % were susceptible to penicillin (Table 1). Erythromycin-induced resistance to clindamycin was not detected in any of the MSSA or MRSA isolates.

**Table 1:** Antibiotic susceptibility of methicillin-sensitive and methicillin-resistant *Staphylococcus aureus*

	Susceptibility [Frequency (%)]			
Drug (AC^a^ μg)	MSSA^b^ (*N* = 151)		MRSA^c^ (*N* = 20)	
Cefoxitin (30)	151	(100)	0	0
Clindamycin (2)	149	(98.7)	20	(100)
Cotrimoxazole (30)	142	(94.0)	17	(85.0)
Erythromycin (15)	114	(75.5)	12	(60.0)
Penicillin (10)	11	(7.3)	0	0

^a^AC: antibiotic concentration

^b^Methicillin-sensitive *Staphylococcus aureus*

^c^Methicillin-resistant *Staphylococcus aureus*

The prevalence of MRSA carriage was equal among groups of the same age, and increased with age (Table 2). Overall, nasal carriage of *S. aureus* was more common in women (12.7 %) than in men (8.0 %). Carriage of *S. aureus* and MRSA was slightly higher in HCW that reported direct patient contact (Table 3). *S. aureus* and MRSA carriage stratified according to medical profession or workplace is shown in Table 3. There was no strong association between MRSA carriage and other risk factors (Tables 4 and 5). Multivariable logistic regression analyses identified female gender and age > 25 years as significantly associated with nasal carriage of *S. aureus* (*p* = 0.003 and *p* = 0.04, respectively) (Table 6).

**Table 2:** Prevalence of *Staphylococcus aureus* (*S. aureus*) and methicillin-sensitive (MSSA) and methicillin-resistant *Staphylococcus aureus* (MRSA) in healthcare workers and students in Antananarivo, Madagascar (*N* = 1548)

		Healthcare workers^a^						Students					
			*S. aureus*	MSSA		MRSA			*S. aureus*	MSSA		MRSA	
		Total number (N)	n (%)	n (%)	*p* ^*b*^	n (%)	*p* ^*b*^	Total number (N)	n (%)	n (%)	*p* ^*b*^	n (%)	*p* ^*b*^
Prevalence		863	91 (10.4)	77 (8.9)	--	14 (1.5)	--	685	80 (11.8)	74 (10.8)	--	6 (0.9)	--
Characteristics													
Age													
	<25 years	403	33 (8.2)	29 (7.2)	0.11	4 (1.0)	0.19*	603	70 (11.5)	65 (10.8)	0.80	5 (0.7)	0.52*
	≥25 years	452	56 (12.4)	46 (10.2)		10 (2.1)		78	10 (12.7)	9 (11.4)		1 (1.3)	
Sex													
	Female	580	72 (12.3)	60 (10.2)	0.04	12 (2.1)	0.16*	440	57 (12.6)	52 (11.7)	0.24	5 (1.0)	0.43*
	Male	283	19 (6.6)	17 (5.1)		2 (0.7)		245	23 (9.4)	22 (9.0)		1 (0.3)	

^a^Includes all hospital employees (medical doctors, nurses, midwives, nursing students, medical and pharmacy students, interns, occasional workers, medical technologists, pharmacists, cleaning staff, and receptionists)

^b^Pearson’s χ^2^ test or * Fisher’s exact test

**Table 3:** Descriptive characteristics of healthcare workers from Antananarivo, Madagascar carrying *Staphylococcus aureus (S. aureus)* and methicillin-resistant *Staphylococcus aureus* (MRSA)

	Healthcare workers^a^ (*n* = 863)		
		*S. aureus*	MRSA
Variable	HCW^b^ group (n)	n (%)	n (%)
Contact with patients	746	81 (10.9)	14 (1.9)
No contact with patients	105	7 (6.7)	0
Type of healthcare worker^c^			
Physician	183	13 (7.1)	1 (0.6)
Nurse	203	23 (11.2)	4 (2.0)
Medical technologist	18	3 (16.7)	2 (11.1)
Other (clerk, service assistant)	9	1 (11.0)	0
Work place^c^			
Outpatient department	5	3 (60.0)	0
Operating theatre	6	1 (16.7)	0
Ward	87	11 (12.5)	2 (2.3)
Other (administration)	52	2 (3.9)	0

^a^ Includes all hospital employees: medical doctors, nurses, midwives, nursing students, medical and pharmacy students, interns, occasional workers, medical technologists, pharmacists, cleaning staff, and receptionists

^b^ Healthcare worker

^c^ For available data

**Table 4:** Descriptive characteristics of healthcare workers from Antananarivo, Madagascar, with respect to carriage of *Staphylococcus aureus* (*S. aureus*) and methicillin-resistant *Staphylococcus aureus* (MRSA)

		Healthcare workers^a^ (*n* = 863)				
			*S. aureus*		MRSA	
Variables	Total number (N^b)^	HCW^c^ group (n)^b^	n (%)	*p* ^*d*^	n (%)	*p* ^*d*^
Previous hospitalisation	47	37	1 (2.7)	0.17*	0	1.0*
No previous hospitalisations	1479	816	89 (10.8)		14 (1.7)	
Previous antimicrobial use^e^	493	310	27 (8.7)	0.18	4 (1.3)	0.59*
No previous antimicrobial use	919	506	59 (11.7)		10 (2.0)	
Unknown	108					
Skin infection	214	121	15 (12.4)	0.47	3 (2.5)	0.44*
No skin infection	1300	724	74 (10.2)		11 (1.5)	
Chronic illness^f^	119	68	9 (13.1)	0.44	0	0.62*
No chronic illness	1358	772	79 (10.2)		14 (1.8)	
Nursing at home^g^	486	403	41 (10.2)	0.73	7 (1.7	0.84
No nursing at home	1025	449	49 (10.9)		7 (1.6)	
Living in dormatory	190	94	7 (7.5)	0.30	0 (1.1)	0.37*
Not living in dormatory	1306	757	82 (10.8)		14 (2.7)	
Contact with animals ^h^	764	417	41 (9.8)	0.50	7 (1.7)	0.90
No contact with animals	784	446	50 (11.2)		7 (1.6)	

^a^ Includes all hospital employees: medical doctors, nurses, midwives, nursing students, medical and pharmacy students, interns, occasional workers, medical technologists, pharmacists, cleaning staff, and receptionists

^b^ For available data

^c^ Healthcare worker

^d^ Pearson’s χ^2^ test or * Fishers exact test

^e^During the last 6 months

^f^ Chronic illness affecting the immune system

^g^ Nursing sick family members at home

^h^ Contact with pets and/or livestock such as cattle, poultry, pigs, sheep and goats

**Table 5:** Descriptive characteristics of students from Antananarivo, Madagascar, with respect to carriage of *Staphylococcus aureus* (*S. aureus*) and methicillin-resistant *Staphylococcus aureus* (MRSA)

		Students (*n* = 685)				
			*S. aureus*		MRSA	
Variables	Total number (N^a^)	Student group (n)^a^	n (%)	*p* ^*b*^	n (%)	*p* ^*b*^
Previous hospitalisation	47	10	2 (20.0)	0.34*	0	1.0*
No previous hospitalisations	1479	663	78 (11.9)		6 (0.9)	
Previous antimicrobial use^c^	493	183	19 (10.4)	0.99	1 (0.6)	1.0*
No previous antimicrobial use	919	413	43 (10.4)		4 (1.0)	
Unknown	108					
Skin infection	214	93	5 (5.4)	0.04*	0	1.0*
No skin infection	1300	576	75 (13.0)		6 (1.0)	
Chronic illness^d^	119	51	8 (15.7)	0.37	0	1.0*
No chronic illness	1358	586	67 (11.3)		4 (0.7)	
Nursing at home^e^	486	83	9 (10.8)	0.77	0	1.0*
No nursing at home	1025	576	69 (12.0)		6 (1.0)	
Living in dormatory	190	96	8 (8.2)	0.28	0	0.60*
Not living in dormatory	1306	549	67 (12.2)		6 (1.1)	
Contact with animals ^f^	764	347	41 (11.8)	0.91	2 (0.6)	0.43*
No contact with animals	784	338	39 (11.4)		4 (1.2)	

^a^For available data

^b^ Pearson’s χ^2^ test or * Fishers exact test

^c^During the last 6 months

^d^ Chronic illness affecting the immune system

^e^ Nursing sick family members at home

^f^ Contact with pets and/or livestock such as cattle, poultry, pigs, sheep and goats

**Table 6:** Multivariable logistic regression analyses of factors associated with nasal colonisation of healthcare workers and university students by *Staphylococcus aureus*

Variable	Category	n	OR^a^ (95 % CI^b^)	*p*
Age				
	Age <25 (years)	403	1	
	Age >25 (years)	452	1.47 (1.02–2.13)	0.04
Sex				
	Female	580	1	
	Male	283	0.57 (0.40–0.83)	0.003
Occupation				
	Student	685	1	
	Healthcare worker	863	0.77 (0.54–1.11)	0.16
Antibiotics^c^				
	No previous antimicrobial use	919	1	
	Previous antimicrobial use	493	1.17 (0.91–1.49)	0.22

^a^OR, odds ratio, adjusted according to multivariable logistic regression

^b^CI, confidence interval

^c^During the last 6 months

In total, 21 *S. aureus* isolates (11.7 %) harboured PVL and 36 (21.1 %) harboured TSST-1. Ten (1.2 %) HCW and 11 (1.6 %) students carried PVL-positive strains in the anterior nares, while 20 HCW (2.3 %) and 16 (1.9 %) students carried TSST-1-positive strains (Table 7).

**Table 7:** Molecular epidemiology of methicillin-resistant *Staphylococcus aureus* isolated from students and healthcare workers from Antananarivo (*n* = 20)

ST^a^	CC^b^	*spa* type^c^	PVL^+^ (n) ^d^	TSST-1^+^ (n) ^e^	Healthcare workers (n)	Students (n)
88^f^	88^g^	t186	0	1	12	4
88^g^	88^g^	t2393	0	0	0	1
n.d^h^	n.d^h^	t5562	0	0	0	1
n.d^h^	n.d^h^	t5772	0	0	1	0
n.d^h^	n.d^h^	t13653	0	1	1	0

^a^ Sequence type

^b^ Clonal complex

^c^ *Staphylococcal* protein A

^d^ Panton-Valentine leukocidin gene present

^e^ Toxic shock syndrome toxin gene present

^f^ Automatically allocated by the Ridom SpaServer [25]

^g^ Retrieved from the literature [37]

^h^ Not determined

Recent findings indicate global occurrence of a new divergent *mecA* homologue, named *mecC* [29]; therefore, all samples were also screened for the *mecC* gene. All MRSA isolates identified in this study harboured the *mecA* gene, but not the *mecC* gene.

*Staphylococcus* protein A gene (*spa*) typing was performed for all 20 MRSA isolates. Of the five different *spa* types identified, *spa* type t186 was the predominant clone (16/20, 80.0 %) among both HCW and students (Table 7). The associated sequence type (ST) for *spa* types t186 and t2393 was ST-88. Eighteen MRSA-positive participants were treated with Mupirocin and were successfully decolonised. In accordance with the current guidelines of the Robert-Koch-Institute, Germany, one HCW required repeated Mupirocin treatment to ensure eradication [28].

## Discussion

To the best of our knowledge, this is the first study to report nasal *S. aureus* and MRSA carriage rates in HCW and non-medical university students in Madagascar. The overall prevalence of nasal *S. aureus* carriage identified in this study is lower than that reported in another study from Madagascar, which identified MSSA in 38 % of outpatients [7].

The finding that nasal carriage of *S. aureus* is higher in females is in line with a study of HCW from Norway, which showed that female HCW had a 54 % increased risk of *S. aureus* nasal carriage *versus* non-HCW (OR, 1.54; 95 % CI, 1.09–2.19). However, the Norwegian study differed from the current study in terms of both study design and detection methods used [30].

Data regarding nasal carriage of MRSA by African HCW are scarce. Studies of HCW from Ethiopia and Libya identified nasal carriage rates for MRSA of 14.1 % [31] and 36.8 % [32]), respectively, whereas a study from Kenya found a nasal carriage rate of 0 % [33]. A study from São Tomé and Principe sampled HCW at two different time points and found that the MRSA prevalence ranged from 1.6 to 4.0 % [34]. A systematic review of MRSA carriage by HCW in non-outbreak situations in Europe and the USA calculated a pooled MRSA colonisation rate of 1.8 % (95 % CI, 1.34–2.50 %) [35]. Carriage rates identified by the individual studies in this particular review ranged from 0.2 to 15 %. Taken together, these studies show that MRSA carriage rates in HCW are highly variable.

PVL is a cytotoxin that causes dermonecrosis and lysis of human granulocytes and increases the adherence of *S. aureus* to the extracellular matrix [36]. Evidence suggests that PVL is associated with staphylococcal skin and soft tissue infections and with severe necrotising pneumonia [18]. As MRSA-colonised HCW may act as a source of infection in outbreak settings [3], we sought to identify this virulence factor in all *S. aureus* isolates. Previous studies from Africa reported high rates for PVL in MSSA strains (ranging from 17 to 74 %) [8, 16]. A study from Gabon reported high PVL rates in both clinical isolates (54.7 %) and isolates obtained from asymptomatic carriers (40.5 %) [21]. Finally, a study by Breurec et al. found that 30 % of clinical isolates from Antananarivo were PVL-positive [8]. The comparatively moderate PVL-positivity rate (11.6 %) identified in our study is in contrast with that found in industrialised countries, in which PVL is rare; however, our findings support those of previous studies from sub-Saharan Africa [8, 16, 21].

A recent study from Congo identified the TSST-1-encoding gene in 17.5 % of all *S. aureus* isolates obtained from HCW [20]. We found even higher rates in both groups examined herein, suggesting that TSST-1 may be more prevalent in *S. aureus* strains from Africa than in strains from Europe, making infection control practices in Malagasy hospitals an urgent priority.

MRSA *spa* type t186 was the predominant clone identified in the current cohort, and the most common type identified in HCW. *Spa* type t186 was one of the clones previously circulating in hospital patients in Antananarivo, Madagascar [15]. According to the RidomSpa Server database [26], this clone occurs worldwide, and the automatically allocated sequence type ST-88 [27] is predominant in West, Central and East Africa [14]. The *spa* type t2393 identified herein was also identified in hospital isolates from Ghana, north-eastern Argentina and from a patient from Berlin with a travel history to Panama, whereas *spa* type t5562 was found in New Zealand isolates [37–40].

### Limitations

A lack of selective enrichment before culture on agar plates may explain the low prevalence of *S. aureus* detection. Also, *S. aureus* frequently colonises other sites on the skin, which were not swabbed in this study due to ethical and operational reasons [41]. This may also account for the low rate of *S. aureus* detection.

The samples examined herein were collected by self-administered nasal swabbing; therefore, sampling may have not been carried out in the best possible way. However, other large, population-based studies of MRSA carriage in Europe were based on self-swabbing techniques, and nasal self-swabbing itself has been shown to be appropriate for the detection of *S. aureus* and MRSA [42–44].

A large part of our study population consisted of students; therefore, we may expect a lower prevalence of *S. aureus* in such healthy participants compared with that in outpatients or hospitalised patients.

It was our intention not to include high-risk groups such as children or people over the age of 69 years, and a group of healthy and relatively young students was used as a proxy for community controls. The relatively low number of *S. aureus* and MRSA isolates mean that the present study has low statistical power; therefore, we may not have detected any association between colonisation and risk factors such as skin lesions [3].

## Conclusion

In conclusion, this study provides a good overview of MRSA clones circulating in this particular region of Madagascar. The prevalence of MRSA in HCW and healthy students from Madagascar was low, and all *S. aureus* strains showed high resistance to penicillin, which is in line with other reports across the African continent. *Spa* type t186, which is the dominant clone on mainland Africa, was also the predominant MRSA clone identified in the current study population. The fairly high rates of TSST-1 and the presence of PVL-producing strains represent a potential reservoir and source for possible severe infection, which could lead to the emergence of successful MRSA clones. From a clinical point-of-view, MRSA screening programs in Africa are limited by a lack of facilities and a lack of qualified staff and financial resources. Antibiotic regimens to eradicate MRSA are expensive. However, general hygiene measures (e.g., hand washing) are both cheap and effective at preventing the spread of MRSA by HCW and should be promoted and performed diligently.

## Abbreviations

BURP, based-upon-repeat-pattern; CC, clonal complex; CLSI, Clinical and Laboratory Standards Institute; HCW, Healthcare workers; MLST, multi-locus sequence type; MRSA, methicillin-resistant *Staphylococcus aureus*; MSSA, methicillin-sensitive *Staphylococcus aureus*; PVL, Panton-Valentine leukocidin; *S. aureus*, *Staphylococcus aureus*; *spa* typing, *Staphylococcus* protein A gene typing; ST, sequence type; TSST, toxic shock syndrome toxin

## Additional file

Additional file 1: Supporting Information **Table S1.** Primers used for PCR screening of nasal *S. aureus* isolates obtained from Madagascan students and healthcare workers. (PDF 73 kb)

## References

[1] Chambers HF, Deleo FR (2009). Waves of resistance: Staphylococcus aureus in the antibiotic era. Nat Rev Microbiol.

[2] Albrich WC, Harbarth S (2008). Health-care workers: source, vector, or victim of MRSA?. Lancet Infect Dis.

[3] Hawkins G, Stewart S, Blatchford O, Reilly J (2011). Should healthcare workers be screened routinely for meticillin-resistant Staphylococcus aureus? A review of the evidence. J Hosp Infect.

[4] Jevons MP, Coe AW, Parker MT (1963). Methicillin resistance in staphylococci. Lancet.

[5] Humphreys H, Grundmann H, Skov R, Lucet JC, Cauda R (2009). Prevention and control of methicillin-resistant Staphylococcus aureus. Clin Microbiol Infect.

[6] Herrmann M, Abdullah S, Alabi A, Alonso P, Friedrich AW, Fuhr G (2013). Staphylococcal disease in Africa: another neglected ‘tropical’ disease. Future Microbiol.

[7] Rasamiravaka T, Rasoanandrasana S, Zafindraibe NJ, Rakoto Alson AO, Rasamindrakotroka A (2013). Evaluation of methicillin-resistant Staphylococcus aureus nasal carriage in Malagasy patients. J Infect Dev Ctries.

[8] Breurec S, Fall C, Pouillot R, Boisier P, Brisse S, Diene-Sarr F (2011). Epidemiology of methicillin-susceptible Staphylococcus aureus lineages in five major African towns: high prevalence of Panton-Valentine leukocidin genes. Clinical microbiology and infection : the official publication of the European Society of Clinical Microbiology and Infectious Diseases.

[9] Randrianirina F, Soares JL, Ratsima E, Carod JF, Combe P, Grosjean P (2007). In vitro activities of 18 antimicrobial agents against Staphylococcus aureus isolates from the Institut Pasteur of Madagascar. Ann Clin Microbiol Antimicrob.

[10] Decousser JW, Pfister P, Xueref X, Rakoto-Alson O, Roux JF (1999). Acquired antibiotic resistance in Madagascar: first evaluation. Med Trop (Mars).

[11] Randrianirina F, Vaillant L, Ramarokoto CE, Rakotoarijaona A, Andriamanarivo ML, Razafimahandry HC (2010). Antimicrobial resistance in pathogens causing nosocomial infections in surgery and intensive care units of two hospitals in Antananarivo, Madagascar. Journal of infection in developing countries.

[12] Uhlemann AC, Otto M, Lowy FD, DeLeo FR (2014). Evolution of community- and healthcare-associated methicillin-resistant Staphylococcus aureus. Infect Genet Evol.

[13] Otter JA, French GL (2010). Molecular epidemiology of community-associated meticillin-resistant Staphylococcus aureus in Europe. Lancet Infect Dis.

[14] Schaumburg F, Alabi AS, Peters G, Becker K. New Epidemiology of Staphylococcus aureus infection from Africa. Clin Microbiol Infect. 2014. 10.1111/1469-0691.12690 24861767

[15] Breurec S, Zriouil SB, Fall C, Boisier P, Brisse S, Djibo S (2011). Epidemiology of methicillin-resistant Staphylococcus aureus lineages in five major African towns: emergence and spread of atypical clones. Clinical microbiology and infection : the official publication of the European Society of Clinical Microbiology and Infectious Diseases.

[16] Ruimy R, Maiga A, Armand-Lefevre L, Maiga I, Diallo A, Koumare AK (2008). The carriage population of Staphylococcus aureus from Mali is composed of a combination of pandemic clones and the divergent Panton-Valentine leukocidin-positive genotype ST152. J Bacteriol.

[17] Fall C, Richard V, Dufougeray A, Biron A, Seck A, Laurent F (2014). Staphylococcus aureus nasal and pharyngeal carriage in Senegal. Clinical microbiology and infection : the official publication of the European Society of Clinical Microbiology and Infectious Diseases.

[18] Shallcross LJ, Fragaszy E, Johnson AM, Hayward AC (2013). The role of the Panton-Valentine leucocidin toxin in staphylococcal disease: a systematic review and meta-analysis. Lancet Infect Dis.

[19] Findlay RF, Odom RB (1982). Toxic shock syndrome. Int J Dermatol.

[20] De Boeck H, Vandendriessche S, Hallin M, Batoko B, Alworonga JP, Mapendo B (2015). Staphylococcus aureus nasal carriage among healthcare workers in Kisangani, the Democratic Republic of the Congo. Eur J Clin Microbiol Infect Dis.

[21] Schaumburg F, Ngoa UA, Kosters K, Kock R, Adegnika AA (2011). Virulence factors and genotypes of Staphylococcus aureus from infection and carriage in Gabon. Clinical microbiology and infection : the official publication of the European Society of Clinical Microbiology and Infectious Diseases.

[22] Paterson GK, Harrison EM, Holmes MA (2014). The emergence of mecC methicillin-resistant Staphylococcus aureus. Trends Microbiol.

[23] Dal-Poz MR. Counting health workers: definitions, data, methods and global results In*.*: World Health Organization; January 2007. http://www.who.int/hrh/documents/counting_health_workers.pdf. Accessed 07 June 2016.

[24] Cheesbrough M. District Laboratory Practice in Tropical Countries, vol. 2. Cambridge: Cambridge University Press; 2005.

[25] Stegger M, Andersen PS, Kearns A, Pichon B, Holmes MA, Edwards G (2012). Rapid detection, differentiation and typing of methicillin-resistant Staphylococcus aureus harbouring either mecA or the new mecA homologue mecA(LGA251). Clinical microbiology and infection : the official publication of the European Society of Clinical Microbiology and Infectious Diseases.

[26] Harmsen D, Claus H, Witte W, Rothganger J, Claus H, Turnwald D (2003). Typing of methicillin-resistant Staphylococcus aureus in a university hospital setting by using novel software for spa repeat determination and database management. J Clin Microbiol.

[27] Mellmann A, Weniger T, Berssenbrugge C, Rothganger J, Sammeth M, Stoye J (2007). Based Upon Repeat Pattern (BURP): an algorithm to characterize the long-term evolution of Staphylococcus aureus populations based on spa polymorphisms. BMC Microbiol.

[28] Robert-Koch-Institut: Empfehlungen zur Prävention und Kontrolle von Methicillin-resistenten Staphylococcus aureus -Stämmen (MRSA) in medizinischen und pflegerischen Einrichtungen. In: Empfehlung der Kommission für Krankenhaushygiene und Infektionsprävention (KRINKO) beim Robert Koch-Institut. Bundesgesundheitsbl. 2014, 57: 696–732. 10.1007/s00103-015-2176-8 25939733

[29] Laurent F, Chardon H, Haenni M, Bes M, Reverdy ME, Madec JY (2012). MRSA harboring mecA variant gene mecC, France. Emerg Infect Dis.

[30] Olsen K, Sangvik M, Simonsen GS, Sollid JU, Sundsfjord A, Thune I (2013). Prevalence and population structure of Staphylococcus aureus nasal carriage in healthcare workers in a general population. The Tromso Staph and Skin Study. Epidemiol Infect.

[31] Gebreyesus A, Gebre-Selassie S, Mihert A (2013). Nasal and hand carriage rate of methicillin resistant Staphylococcus aureus (MRSA) among health care workers in Mekelle Hospital, North Ethiopia. Ethiop Med J.

[32] Zorgani A, Elahmer O, Franka E, Grera A, Abudher A, Ghenghesh KS (2009). Detection of meticillin-resistant Staphylococcus aureus among healthcare workers in Libyan hospitals. The Journal of hospital infection.

[33] Omuse G, Kariuki S, Revathi G (2012). Unexpected absence of meticillin-resistant Staphylococcus aureus nasal carriage by healthcare workers in a tertiary hospital in Kenya. The Journal of hospital infection.

[34] Conceicao T, Santos Silva I, de Lencastre H, Aires-de-Sousa M (2014). Staphylococcus aureus nasal carriage among patients and health care workers in Sao Tome and Principe. Microb Drug Resist.

[35] Dulon M, Peters C, Schablon A, Nienhaus A (2014). MRSA carriage among healthcare workers in non-outbreak settings in Europe and the United States: a systematic review. BMC Infect Dis.

[36] Loffler B, Hussain M, Grundmeier M, Bruck M, Holzinger D, Varga G, Roth J (2010). Staphylococcus aureus panton-valentine leukocidin is a very potent cytotoxic factor for human neutrophils. PLoS Pathog.

[37] Egyir B, Guardabassi L, Sorum M, Nielsen SS, Kolekang A, Frimpong E (2014). Molecular epidemiology and antimicrobial susceptibility of clinical Staphylococcus aureus from healthcare institutions in Ghana. PLoS One.

[38] Gardella N, von Specht M, Cuirolo A, Rosato A, Gutkind G, Mollerach M (2008). Community-associated methicillin-resistant Staphylococcus aureus, eastern Argentina. Diagn Microbiol Infect Dis.

[39] Nurjadi D, Friedrich-Janicke B, Schafer J, Van Genderen PJ, Goorhuis A, Perignon A (2015). Skin and soft tissue infections in intercontinental travellers and the import of multi-resistant Staphylococcus aureus to Europe. Clinical microbiology and infection : the official publication of the European Society of Clinical Microbiology and Infectious Diseases.

[40] Williamson DA, Lim A, Thomas MG, Baker MG, Roberts SA, Fraser JD (2013). Incidence, trends and demographics of Staphylococcus aureus infections in Auckland, New Zealand, 2001-2011. BMC Infect Dis.

[41] Sollid JU, Furberg AS, Hanssen AM, Johannessen M (2014). Staphylococcus aureus: determinants of human carriage. Infection, genetics and evolution : journal of molecular epidemiology and evolutionary genetics in infectious diseases.

[42] Gamblin J, Jefferies JM, Harris S, Ahmad N, Marsh P, Faust SN (2013). Nasal self-swabbing for estimating the prevalence of Staphylococcus aureus in the community. J Med Microbiol.

[43] Akmatov MK, Mehraj J, Gatzemeier A, Strompl J, Witte W, Krause G, Pessler F (2014). Serial home-based self-collection of anterior nasal swabs to detect Staphylococcus aureus carriage in a randomized population-based study in Germany. Int J Infect Dis.

[44] van Cleef BA, van Rijen M, Ferket M, Kluytmans JA (2012). Self-sampling is appropriate for detection of Staphylococcus aureus: a validation study. Antimicrob Resist Infect Contr.

