# Supplementary material for: Prevalence of nasal colonisation by methicillin-sensitive and methicillin-resistant Staphylococcus aureus among healthcare workers and students in Madagascar
Source: BMC Infect Dis. 2016 Aug 15;16:420. doi: 10.1186/s12879-016-1733-6 (PMC4986198; doi:10.1186/s12879-016-1733-6)
Supplement: Additional file 1: — Supporting Information Table S1. Primers used for PCR screening of nasal S. aureus isolates obtained from Madagascan students and healthcare workers. (PDF 73 kb) [file 12879_2016_1733_MOESM1_ESM.pdf]

Supporting Information Table 1. Primers used for PCR screening of nasal *S. aureus* isolates obtained from Madagascan students and healthcare workers.

Primer	Target gene	PCR product size (bp[a])	Sequence 5' to 3'	References
nuc1	nuc	279	GCG ATT GAT GGT GAT ACG GTT	Zhang K et al 2004[1]
nuc2			AGC CAA GCC TTG ACG AAC TAA AGC	
$mecA_{LGA251}$	FP $mecA_{LGA251}$	356	TCACCAGGTTCAAC[Y]CAAAA	Stegger M et al 2012[2]
$mecA_{LGA251}$RP			CCTGAATC[W]GCTAATAATATTTC	
pvl-FP	lukF-PV	83	GCTGGACAAAACTTCTTGGAATAT	Stegger M et al 2012[2]
pvl-RP			GATAGGACACCAATAAATTCTGGATTG	
Tst-F	TSST-1	445	AAG CCC TTT GTT GCT TGC G	Becker K et al 1998[3]
Tst-R			ATC GAA CTT TGG CCC ATA CTT T	
MecAP4	MecA	162	TCCAGATTACAACTTCACCAGG	Oliveira DC et al 2002[4]
MecAP7			CCACTTCATATCTTGTAACG	
MecC-F	Mec_{LGA251}	304	GCTCCTAATGCTAATGCA	Cuny C et al 2011[5]
MecC-R			TAAGCAATAATGACTACC	

[a]Base pairs.

References

1. Zhang K, Sparling J, Chow BL, Elsayed S, Hussain Z, Church DL, et al. New quadriplex PCR assay for detection of methicillin and mupirocin resistance and simultaneous discrimination of Staphylococcus aureus from coagulase-negative staphylococci. *Journal of clinical microbiology* 2004;42(11):4947-55.
2. Stegger M, Andersen PS, Kearns A, Pichon B, Holmes MA, Edwards G, et al. Rapid detection, differentiation and typing of methicillin-resistant Staphylococcus aureus harbouring either mecA or the new mecA homologue mecA(LGA251). *Clinical microbiology and infection: the official publication of the European Society of Clinical Microbiology and Infectious Diseases* 2012;18(4):395-400.
3. Becker K, Roth R, Peters G. Rapid and specific detection of toxigenic Staphylococcus aureus: use of two multiplex PCR enzyme immunoassays for amplification and hybridization of staphylococcal enterotoxin genes, exfoliative toxin genes, and toxic shock syndrome toxin 1 gene. *Journal of clinical microbiology* 1998;36(9):2548-53.
4. Oliveira DC, de Lencastre H. Multiplex PCR strategy for rapid identification of structural types and variants of the mec element in methicillin-resistant Staphylococcus aureus. *Antimicrobial agents and chemotherapy* 2002;46(7):2155-61.
5. Cuny C, Layer F, Strommenger B, Witte W. Rare occurrence of methicillin-resistant Staphylococcus aureus CC130 with a novel mecA homologue in humans in Germany. *PloS one* 2011;6(9):e24360.
